# Zigzag boron nitride nanoribbon doped with carbon atom for giant magnetoresistance and rectification behavior based nanodevices

**DOI:** 10.1038/s41598-024-62721-9

**Published:** 2024-06-19

**Authors:** Rigao Wang, Feng Shuang, Mingsong Lin, Xiangfu Wei, Zheng Fang, Duan She, Wei Cai, Xiaowen Shi, Mingyan Chen

**Affiliations:** 1 Guangxi Key Laboratory of Intelligent Control and Maintenance of Power Equipment, School of Electrical Engineering, Guangxi University, Nanning, 530004 China; 2 Guangxi Vocational and Technical College of Communications, Nanning, 530023 China; 3 Institute of Physics, Henan Academy of Sciences, Zhengzhou, 450046 China; 4 Hongzhiwei Technology (Shanghai) Co. Ltd., 1599 Xinjinqiao Road, Pudong, 201206 Shanghai China

**Keywords:** Electronic and spintronic devices, Magnetic devices

## Abstract

Using the principles of density functional theory (DFT) and nonequilibrium Green’s function (NEGF), We thoroughly researched carbon-doped zigzag boron nitride nanoribbons (ZBNNRs) to understand their electronic behavior and transport properties. Intriguingly, we discovered that careful doping can transform carbon-doped ZBNNRs into a spintronic nanodevice with distinct transport features. Our model showed a giant magnetoresistance (GMR) up to a whopping 10^5^ under mild bias conditions. Plus, we spotted a spin rectifier having a significant rectification ratio (RR) of 10^4^. Our calculated transmission spectra have nicely explained why there’s a GMR up to 10^5^ for spin-up current at biases of $-1.2$ V, $-1.1$ V, and $-1.0$ V, and also accounted for a GMR up to 10^3^–10^5^ for spin-down current at biases of 1.0 V, 1.1 V, and 1.2 V. Similarly, the transmission spectra elucidate that at biases of 1.0 V, 1.1 V, and 1.2 V for spin-up, and at biases of 1.1 V and 1.2 V for spin-down in APMO, the RRs reach 10^4^. Our research shines a light on a promising route to push forward the high-performance spintronics technology of ZBNNRs using carbon atom doping. These insights hint that our models could be game-changers in the sphere of nanoscale spintronic devices.

## Introduction

IT is the foundation of modern society’s progress, and there’s a growing demand for faster, more energy-efficient tech in this field. Spintronics, which uses the electron’s spin for new electronic devices, has come up as a cutting-edge approach^1^. It could make data storage and retrieval up to a thousand times faster than current electronics^2^. That’s why finding materials that work well with electron spin is a big deal in research right now.

Recently, researchers have shown a lot of interest in boron nitride nanoribbons (BNNRs), a material known for its special features like outstanding chemical and heat resistance^3,4^. A wide variety of nanodevices have been created from this material. Yet, to broaden the range of these nanodevices, we’re dealing with some issues that need to be resolved, so we’re on the lookout for the right methods to fine-tune its electronic properties^5–10^. A lot of factors can alter BNNRs properties, both structural and spin-polarized,the width and edge shapes^11,12^, edge passivation^12–18^, defects^19–22^, heterostructures^23,24^, doping^25–32^, and so on^33^. The scope of *h*-BN’s potential in spintronics is expanded by its compatibility with various adsorbents and dopants^34^. Y. Liu and colleagues have investigated *h*-BN doped with silicon^34^. Their findings indicate that the silicon atoms induce significant lattice distortions in BN, resulting in the bending of the *h*-BN sheet. Additionally, the introduction of Si dopants leads to two spin-localized states within the band gap of BN, with a magnetic moment centered mainly around the Si dopant, amounting to 1.0 $\mu$B. In separate research based on DFT calculations, D. S. Fartab and team probed the magnetism of *h*-BN when doped with lithium. The optimized structure of the BN sheet exhibited pronounced local distortions^35^. R. Q. Wu and colleagues extended this research to BN nanotubes doped with carbon. Their computational studies show that carbon doping can induce spin polarization^36^, with the magnetic moments primarily arising from the carbon’s 2*p* electrons.

Researchers have been trying to understand the transport properties of ZBNNRs, specifically those that have been edge-Hydrogenated, edge-Fluorinated, and Zn-passivated^13–15^. Rakesh and his team conducted research that revealed insights into the structural, electronic, and quantum transport properties of hydrogenated cove-edge defective ZBNNRs^19^. S. Choudhary and his team have reported findings from first-principles calculations on the spin-related quantum transport in Fe-SiCNT-Fe magnetic tunnel junctions, showing excellent spin filtering and a strong tunnel magnetoresistance^37^. They also explored how boron nitride doped with cobalt behaves in transport, which turned out to be much better than the former^38^.

Despite extensive research into the electronic structure and magnetic properties of ZBNNRs, there’s been a surprising shortage of studies exploring the spin transport properties of those doped with other elements. To fill this gap, we’ve delved into the properties of spin-polarized structural and electronic transport in ZBNNRs doped with a carbon atom. This exploration includes both RR and GMR, both of which we’ve found to be dependent on the spin orientations of opposing electrodes^14^. With our calculated GMR reaching up to 10^5^ and a significant RR magnitude of 10^4^ observed, our models demonstrate an exceptional performance that positions them as strong candidates for spintronics applications.

## Methods

Our calculations were carried out by using *ab* initio software package, DS-PAW, the DFT as executed in the software package to calculate the geometry and electronic properties^39^. The electron-ion potential is depicted using the Projector-Augmented-Wave method^39^, while the electron-electron interactions are handled within the Generalized Gradient Approximation (GGA) framework with the help of the Perdew–Burke–Ernzerhof (PBE) scheme for the exchange-correlation functional^40^. We’ve set the cutoff energy at 500 eV and a 100 $\times 1 \times 1$ *k*-point mesh for the initial Brillouin-zone integration and single-zeta polarized basis set is adopted for electron wave function^41^. We have also ensured the atomic positions are fully relaxed until the remaining force on each atom doesn’t exceed 0.01 eV/Å. In an attempt to minimize the interaction between neighboring units, we’ve established the vacuum spaces along the *y* and *z* axes to be more than 35 Å and 15 Å, respectively.

We’ve simulated the quantum transport properties using the NEGF framework, in tandem with DFT, as deployed in the NanoDCAL software suite^42–44^. The PBE iteration of the GGA was selected for the exchange and correlation functional^45^. We achieved a self-consistent Hamiltonian when the observed measures, such as every component of the Hamiltonians and density matrices, showed a variance of less than $1 \times 10^{-4}$ eV between two consecutive steps of iteration. An energy cutoff of 160 Ry defined the real-space grid. For Brillouin zone sampling, we employed a 100 $\times 1 \times 1$ *k*-point with 20 Å of vacuum spacing.The temperature of the electrodes is set to be 300 K. Both of these statements overview the strategies and methodologies utilized to refine the device structure and compute its transport properties using sophisticated computational tools and methodologies. Using the Landauer-Büttiker formula with a two-probe device, we computed the transport properties^37,46^:

1 $$\begin{aligned} G_{C}^{\uparrow (\downarrow )}(E,V)=\left[ E^{\uparrow (\downarrow )}I-H^{\uparrow (\downarrow )}-\Sigma _{L}^{r}E^{\uparrow (\downarrow )}-\Sigma _{R}^{r}E^{\uparrow (\downarrow )}\right] ^{-1} \end{aligned}$$

2 $$\begin{aligned} T^{\uparrow (\downarrow )}(E,V)=Tr\left[ \tau _{R}\left( E,V\right) G_{C}\left( E,V\right) \tau _{L}\left( E,V\right) G_{C}^{\dagger }\left( E,V\right) \right] ^{\uparrow (\downarrow )} \end{aligned}$$

3 $$\begin{aligned} I^{\uparrow (\downarrow )}\left( V\right) =\frac{e}{h}\int _{\varepsilon L}^{\varepsilon _{R}}T^{\uparrow (\downarrow )}\left( E,V\right) \left[ f_{L}\left( E-\varepsilon _{L}\right) -f_{R}\left( E-\varepsilon _{R}\right) \right] \end{aligned}$$

The advanced and retarded Green’s functions of the scattering region are represented as ${G_C^{\uparrow (\downarrow )}} (E, V )$ and $G_C^\dag (E, V )^{\uparrow (\downarrow )}$, respectively. $\tau _{L(R)}$ signifies the coupling factors of the left/right contact. The unit matrix and the Hamiltonian matrix for the retarded Green’s function are denoted by *I* and $H^{\uparrow (\downarrow )}$, respectively. The terms $\Sigma _L^r$ and $\Sigma _R^r$ correspond to the left and right electrode’s self-energy, respectively, indicating the coupling between the two terminals. The transmission spectrums and the equilibrium Fermi distribution function are represented by $T^{\uparrow (\downarrow )}(E, V)$ and $f_{L(R)}$, respectively. Lastly, $\varepsilon _L$ and $\varepsilon _R$ stand for the electrochemical potentials of the two electrodes, respectively.

## Results and discussion

**Figure 1:**
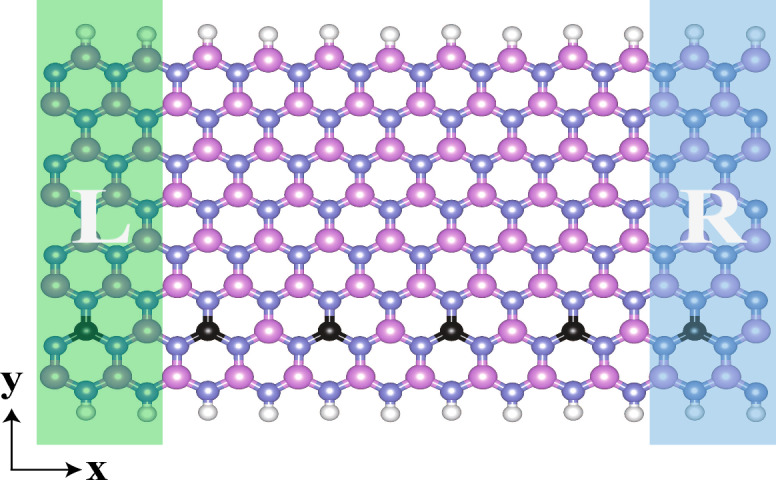
This is the schematic of our model, which is based on Carbon doping in impure ZBNNRs. The pink, blue, black, and white spheres represent boron, nitrogen, carbon, and hydrogen atoms, respectively.

**Figure 2:**
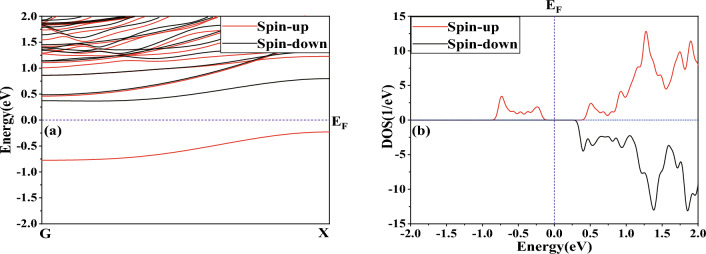
Spin-compensated band structures and DOS of our model. (**a**) Band structures, (**b**) DOS. The violet dashed line at 0 eV represents the Fermi level.

Figure 1 displays a diagram of our model, which is based on Carbon doping in ZBNNRs. Our model’s two electrodes are embodied by the unit cells. The scattering region is achieved by duplicating four unit cells, each doped with a carbon atom, along the transport direction. Initially, we investigated the electronic properties of the Carbon-doped unit cell of ZBNNRs, including their band structures and density of states (DOSs), as seen in Fig. 2. Importantly, as shown in Fig. 2a, two bands are present near the Fermi level, one spin-up (SU) band and one spin-down (SD) band, suggesting significant potential for use in nanoscale spintronic devices. Figure 2b presents the DOS plot of our model, with peaks near the Fermi level primarily consisting of SU electrons. The violation of spin degeneracy between SU and SD electrons near the Fermi level results in the separation of SU and SD energy bands, as visualized in Fig. 2a. This effect is also noticeable in the spin-polarized DOS profile shown in Fig. 2b.

**Figure 3:**
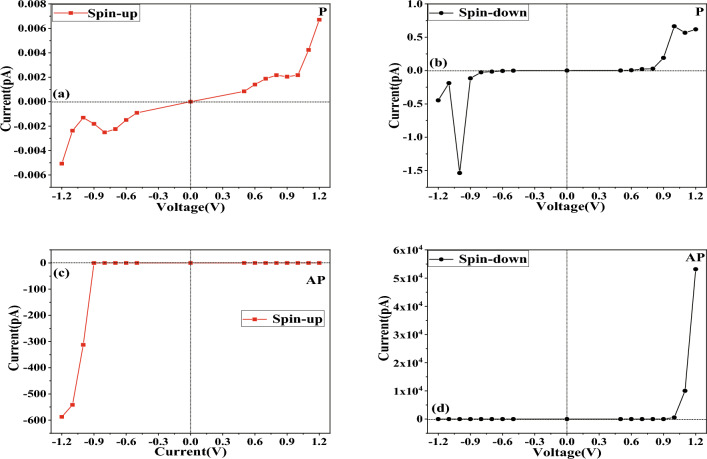
Spin-polarized *I*–*V* characteristics of our model for (**a**) SU and (**b**) SD in PMO, (**c**) SU and (**d**) SD in APMO, respectively.

Following our initial analysis, we explored the quantum transport properties of carbon-doped ZBNNRs in more detail. We were particularly interested in examining the transport characteristics of our proposed model under parallel magnetization orientation (PMO) and antiparallel magnetization orientation (APMO) conditions. We used external magnetic fields to control the spin orientations of the left and right electrodes, denoted as $M_L$ and $M_R$ respectively, as referenced in prior studies by Kharwar. Two configurations were considered in our study: [$M_L$, $M_R$] = [1, -1] and [$M_L$, $M_R$] = [1, 1]^14,47^. The [$M_L$, $M_R$] = [1, -1] configuration denotes an APMO state, with the left and right electrodes having opposite spins, while the [$M_L$, $M_R$] = [1, 1] configuration illustrates PMO, where both electrodes have the same spin. The spin-dependent current-voltage (*I*–*V*) curves of the device are illustrated in Fig. 3. Given that the band gap for SU electrons is 0.6892 eV in the band structure, we chose the bias voltage range of [$-1.2$ V, $-0.5$ V] and [0.5 V, 1.2 V] for our investigation.

**Figure 4:**
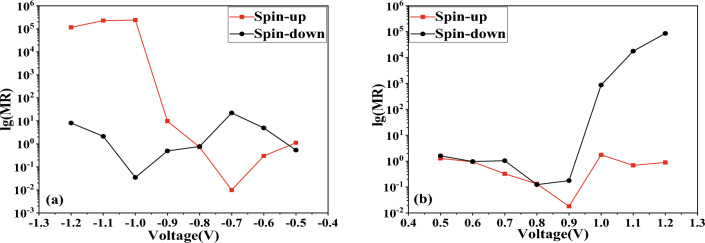
Our model exhibits the base-10 logarithm of the spin-polarized MR for the device under negative (**a**) and positive (**b**) voltages.

**Figure 5:**
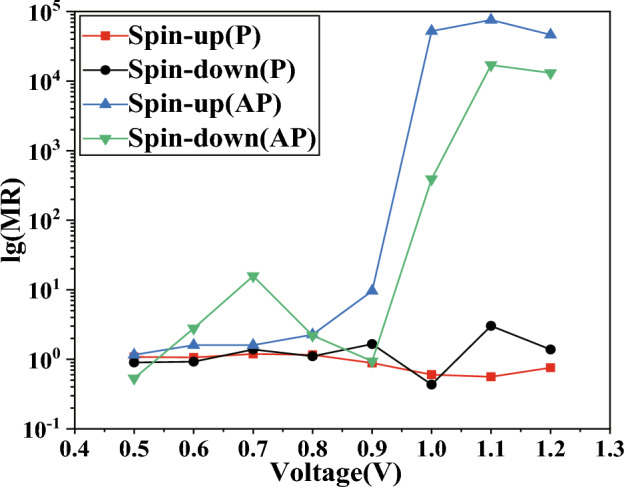
The base-10 logarithm of the spin-polarized RR for our model.

As demonstrated in Fig. 3c, d, in our model, within the framework of APMO, the SD current is stronger at biases of 1.1 V and 1.2 V, while the SU current is more dominant at biases of $-1.0$ V, $-1.1$ V, and $-1.2$ V. The strong current we saw in the device when tested with APMO suggests that the GMR effect is present in our model. Further, we calculated the magnetoresistance (MR) at both positive and negative bias voltages using the following formula^48^:

4 $$\begin{aligned} MR (I_P>I_{AP}) = \frac{I_P-I_{AP}}{I_{AP}} \end{aligned}$$

5 $$\begin{aligned} MR ( I_{AP}>I_P)= \frac{I_{AP}-I_P}{I_P} \end{aligned}$$

The spin-magnetized current in P/AP orientation is indicated as *I*_P_/*I*_AP_, and the common logarithm of MR for both devices is depicted in Fig. 4. The peak MR was marked at $2.3857 \times 10^5$ and 8.5956 $\times 10^4$ at $-1.0$ V and 1.2 V, respectively, attributable to the SU current and SD current, which are comparable in scale to the MR values for zigzag graphene nanoribbons researched by Sun et al.^49^. In the supplementary material, we present the 8-ZGNR device in Fig. S1 and have calculated its *I*–*V* curves, illustrated in Fig. S2. These results are consistent with those of the author. Adhering to the procedures outlined in the original manuscript, we examined the transmission spectra under a bias of 0.01 V for both PMO and APMO conditions. In the PMO case, the transmission spectrum’s peak height is about 0.9834, as depicted in Fig. S3a, whereas in the APMO scenario, it significantly reduces to the order of 10^-6^, as shown in Fig. S3b. Thus, the MR, calculated from the ratio of the areas under the two transmission peaks reaches a significant value of 10^5^. Additionally, we calculated the spin-dependent RR for the SD current ($I_{dn}$) as outlined in the referenced study^50^,

$$\begin{array}{l} RR = \frac{{\left| {I_{dn}(+V)} \right| }}{{\left| {I_{dn}(-V)} \right| }}\quad \quad \quad \quad \quad \quad \quad \quad \quad \quad \quad \quad \quad \quad \quad \quad \quad \quad \quad \quad \quad \quad \quad \quad \quad \quad \quad \quad \quad \quad \quad \quad \quad \quad \quad \quad \quad \quad \quad \quad (6a)\\ \end{array}$$

While the RR for SU current ($I_{su}$) is defined as

$$\begin{array}{l} RR = \frac{{\left| {I_{up}(-V)} \right| }}{{\left| {I_{up}(+V)} \right| }}\quad \quad \quad \quad \quad \quad \quad \quad \quad \quad \quad \quad \quad \quad \quad \quad \quad \quad \quad \quad \quad \quad \quad \quad \quad \quad \quad \quad \quad \quad \quad \quad \quad \quad \quad \quad \quad \quad \quad \quad (6b)\\ \end{array}$$

**Figure 6:**
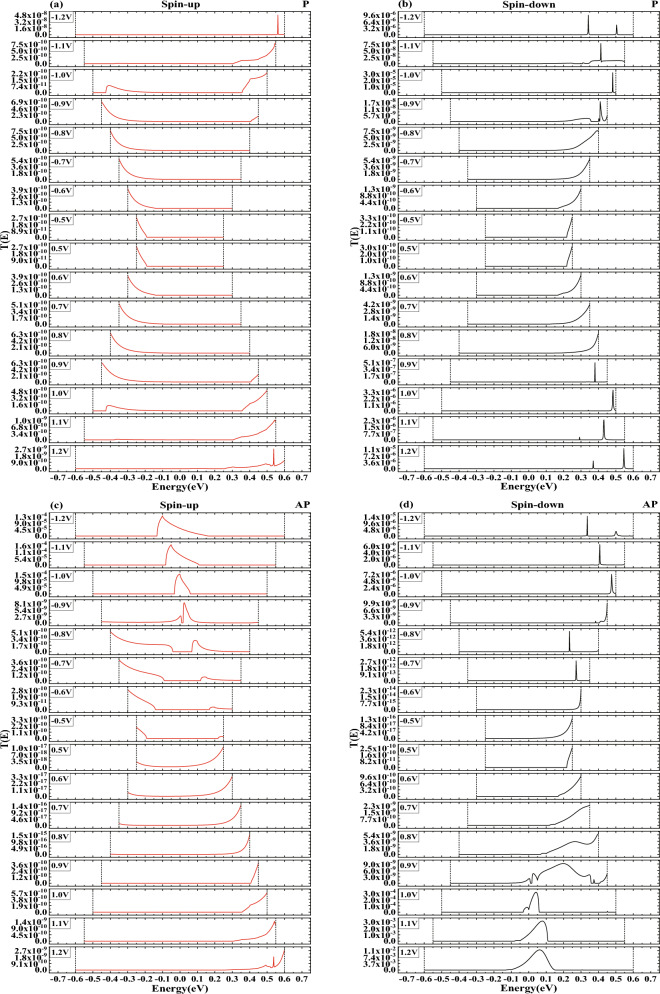
The spin-polarized transmission spectra are provided for: (**a**) SU and (**b**) SD in PMO, (**c**) SU and (**d**) SD in APMO.

Refer to Fig. 5 for exploration of the rectification behavior. The device’s peak RR clocks in at $7.57\times 10^4$, which is two orders of magnitude higher than the RR for zigzag silicene nanoribbons studied by Zhang et al. and edge-defected zigzag silicene nanoribbons investigated by Li et al.^48,51^. To enhance the credibility of the comparison, we selected the H2-5ZSiNR-H device, depicted in Fig. S4. In the supplementary materials, we computed the *I*–*V* curves, as illustrated in Fig. S5, and the transmission spectra under various bias voltages, shown in Fig. S7, in the AP spin configuration. In Fig. S6, we observed that the RR reaches 10^3^ for spin-down electrons when the bias voltage ranges from 0.25 to 0.5 V. In the transmission spectrum, there are almost no transmission peaks for spin-down electrons from -0.5V to 0.1V. However, at a bias voltage of 0.15V to 0.2V, the peak heights of the spin-down electron transmission spectrum are on the order of 10^-5^ to 10^-3^, and at a bias voltage of 0.25V to 0.5V, they reach the order of 10^-1^, with a significantly larger transmission window, which well explains the rectification rate. From the $I-V$ characteristics curve, it is evident that the spin-down current ranges from 0.325 to 2.34 $\upmu$A when the bias voltage is from 0.25 to 0.5 V, and from 0.0708 to 1.03 nA when the bias voltage is from $-0.25$ to $-0.5$ V. In our model, the spin-down current is 10.022 nA and 53.167 nA at bias voltages of 1.1 V and 1.2 V, respectively, and 0.589 pA and 4.053 pA at $-1.1$ V and $-1.2$ V, respectively. Thus, our model exhibits a RR that is an order of magnitude higher than the former. Analyzing Fig. 3 reveals that the SU current component significantly influences the *I*–*V* characteristics of the device in APMO.

To gain a deeper understanding of the GMR and RR phenomena in question, we conducted an analysis of the transmission spectra for ZBNNRs doped with carbon, under various applied voltages in both PMO and APMO, as shown in Fig. 6. As depicted in Fig. 6a, for carbon-doped ZBNNRs in PMO at both negative and positive bias voltages, the electron transmission probability is quite low due to the absence of significant transmission peaks. So, the blue line with upward-pointing triangles in Fig. 5 shows the RR, which matches up with the red squares in that figure. The RRs are consistent, registering at an order of magnitude of 10^0^.

In Fig. 6c, the chance that electrons will pass through at positive voltage is pretty low because there are not any major peaks in transmission. But, when the voltage goes from $-0.5$ down to $-0.8$ V, the electron transmission probabilities (ETPs) jump to 10^-10^. The blue line with upward triangles in Fig. 5 shows a RR that’s around 10^0^ times higher. At a $-0.9$ V bias, the ETP is about 10^-9^, and then it increases by a factor of 10^5^ hitting 10^-4^ at voltages of $-1.0$ V, $-1.1$ V, and $-1.2$ V. With these negative voltages, the ETPs are a lot higher because there are noticeable peaks in transmission, unlike at positive biases. So, the blue line with upward triangles in Fig. 5 indicates an RR of up to 10^4^ at biases of 1.0 V, 1.1 V, and 1.2 V.

Compared to the data shown in Fig. 6a, specifically within the APMO, the ETPs at $-1.0$ V, $-1.1$ V, and $-1.2$ V is an order of magnitude higher at 10^-4^ due to significant transmission peaks; these ETPs are considerably larger than those at positive bias voltages. Therefore, as indicated by the red line with squares in Fig. 4a, at the biases of $-1.0$ V, $-1.1$ V, and $-1.2$ V, the MR reaches up to 10^5^. However, as shown in Fig. 6a, c, in the positive bias voltages, because there are no transmission peaks in either the PMO and APMO, the MR as shown by the red line with squares in Fig. 4b, does not exhibit any significant changes, remaining within the order of magnitude between 10^-1^ and 10^1^.

In Fig. 6b, our model exhibits a low ETP under the PMO condition due to the lack of significant transmission peaks. Consequently, as depicted by the black line with dots in Fig. 5, the RRs are noted to be around the level of 10^0^. However, in Fig. 6d, for our model under APMO conditions, the absence of noteworthy transmission peaks at negative bias voltages means the ETPs remain low. Additionally, no peaks are observed between 0.5 and 0.7 V, with a slight peak appearing at the 10^-9^ level only between 0.8 and 0.9 V, which results in maintaining the RR at a magnitude of 1 for biases ranging from 0.5 to 0.9 V. In contrast, at biases of 1.0 V, 1.1 V, and 1.2 V, the ETPs are an order of magnitude higher at 10^-4^, due to more pronounced transmission peaks, which are significantly greater than those at other bias voltages. Therefore, as illustrated by the green line with upward-pointing triangles in Fig. 5, the RR is recorded as reaching heights of 10^2^, 10^4^, and 10^4^ at the respective biases of 1.0 V, 1.1 V, and 1.2 V.

**Figure 7:**
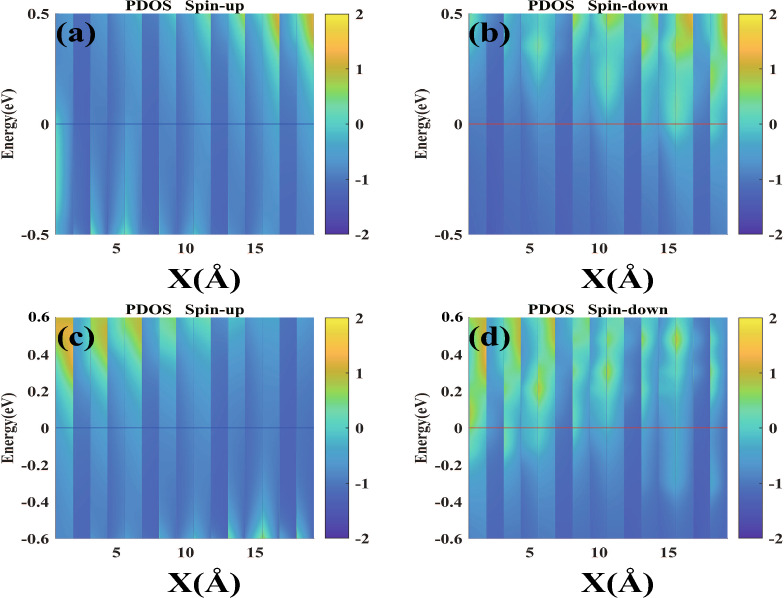
The partial density of states (PDOSs) under two different bias voltages has been calculated: (**a**) SU and (**b**) SD at −1.0 V in PMO, and (**c**) SU and (**d**) SD at 1.2 V in APMO, respectively.

Compared to what we see in Fig. 6b, and specifically within the APMO in Fig. 6d, the absence of transmission peaks in both the PMO and APMO means that the MR at negative bias voltages does not change much. This is illustrated by the black line with dots in Fig. 4a, where the MR stays within a range from 10^-1^ to 10^1^. However, at biases of 1.0 V, 1.1 V, and 1.2 V within the APMO, as shown in Fig. 6d, the MR is an order of magnitude higher at 10^-4^ because there are significant transmission peaks, which are considerably larger than those at positive bias voltages. As a result, as depicted by the black line with dots in Fig. 4b, the MR at these biases increases to 10^3^, 10^4^, and 10^5^, respectively.

Additionally, we showcase the PDOSs at $-1.0$ V for the PMO and at 1.2 V for the APMO for carbon-doped ZBNNRs in Fig. 7. At $-1.0$ V, a review of the SD electrons near the Fermi level in Fig. 7b indicates that the partial density of states for SU electrons near the Fermi level is lower, as seen in Fig. 7a. This is consistent with the observations in Fig. 6a, b, where the ETPs for SU and SD currents are around 10^-10^ and 10^-5^, respectively. Similarly, at 1.2 V, the PDOSs for the APMO are displayed in Fig. 6c, d. Compared to the SD electrons near the Fermi level in Fig. 7d, the SU electrons exhibit a reduced PDOS near the Fermi level, as depicted in Fig. 7c. This correlates with the findings in Fig. 6c, d, where the ETPs for SU and SD currents are roughly 10^-9^ and 10^-2^, respectively.

## Conclusion

In our study, we explored the spin-dependent electronic and transport properties of our model, which is constructed from ZBNNRs doped with carbon atoms, using the DFT-NEGF formalism. Our research reveals that the GMR and RR are of the order of 10^5^ and 10^4^, respectively. These results demonstrate the impressive GMR and RR in our model. Our study further substantiates that the incorporation of carbon atoms markedly affects electron transport in ZBNNRs, offering considerable opportunities for the development of nanoscale devices, particularly in the rapidly advancing field of spintronics. The model under investigation holds wide-ranging potential for a multitude of electronic applications. The observed GMR and rectifying properties render it an excellent candidate for incorporation into spintronic devices such as nano-rectifiers, hard disk read/write heads, Magnetic random access memory, magnetic sensors, among others.

### Supplementary Information

Supplementary Figure S1.

Supplementary Figure S2.

Supplementary Figure S3.

Supplementary Figure S4.

Supplementary Figure S5.

Supplementary Figure S6.

Supplementary Figure S7.

Supplementary Legends.

## Data Availability

All data generated or analysed during this study are included in this published article. The datasets used and/or analysed during the current study available from the corresponding author on reasonable request.
