# Supplementary material for: Zigzag boron nitride nanoribbon doped with carbon atom for giant magnetoresistance and rectification behavior based nanodevices
Source: Sci Rep. 2024 Jun 19;14:14149. doi: 10.1038/s41598-024-62721-9 (PMC11187198; doi:10.1038/s41598-024-62721-9)
Supplement: Supplementary file 8 — Supplementary Legends. [file 41598_2024_62721_MOESM8_ESM.docx]

Figure S1 The diagram provides a simplified view of the device's layout. The parts acting as electrodes are shown in shaded areas. Electrons move in the direction along the x-axis. Along one edge of the device, there are eight oxygen atoms. In this illustration, oxygen atoms are depicted as red spheres, carbon atoms as black, and hydrogen atoms as white.

Figure S2 The spin-dependent I-V curves for 8-ZGNR device configuration with P/AP spin configuration of two electrodes.

Figure S3 The spin-dependent transmission spectrum of 8-ZGNR. (a) P configuration; (b) AP configuration at a low bias voltage of 0.01V.

Figure S4 The device structure with two electrodes constructed from a H2-5ZSiNR-H ribbon. In this design, hydrogen atoms are shown as white spheres, and silicon atoms are depicted as blue spheres. On the top edge of the ribbon, each silicon atom bonds with two hydrogen atoms, while on the bottom edge, each silicon atom bonds with a single hydrogen atom. The images provide two perspectives: (a) a front view and (b) a side view.

Figure S5 Current–voltage (I–V) curves for H2-5ZSiNR-H device for the AP spin configuration, the black and red lines are for the spin-up and spin-down currents, respectively.

Figure S6 (a) and (b) show in a log-scale the rectifying ratio for the spin-up and spin-down current of H2-5ZSiNR-H device, respectively.

Figure S7 The spin-polarized transmission spectra of H2-5ZSiNR-H device in APMO are provided for: (a) spin-up and (b) spin-down.
